# Synergistic Toughening of Epoxy Composite with Cellulose Nanofiber and Continuous Pineapple Leaf Fiber as Sustainable Reinforcements

**DOI:** 10.3390/nano13111703

**Published:** 2023-05-23

**Authors:** Nichapa Klinthoopthamrong, Sombat Thanawan, Gautier Schrodj, Karine Mougin, Kheng-Lim Goh, Taweechai Amornsakchai

**Affiliations:** 1 Polymer Science and Technology Program, Department of Chemistry, Faculty of Science, Mahidol University, Phuttamonthon 4 Road, Salaya, Nakhon Pathom 73170, Thailandsombat.tha@mahidol.ac.th (S.T.); 2 Rubber Technology Research Center, Faculty of Science, Mahidol University, Phuttamonthon 4 Road, Salaya, Nakhon Pathom 73170, Thailand; 3 Institut de Science des Matériaux de Mulhouse, IS2M-CNRS-UHA, 15, Rue Jean Starcky, B.P.2488-68057 Mulhouse, Cedex, France; gautier.schrodj@uha.fr (G.S.); karine.mougin@uha.fr (K.M.); 4 Mechanical Design and Manufacturing Engineering, Newcastle University in Singapore, 172A Ang Mo Kio Avenue 8 #05-01, SIT@NYP Building, Singapore 567739, Singapore; kheng-lim.goh@newcastle.ac.uk; 5 Faculty of Science, Agriculture & Engineering, Newcastle University, Newcastle upon Tyne NE1 7RU, UK; 6 Center of Sustainable Energy and Green Materials, Faculty of Science, Mahidol University, Phuttamonthon 4 Road, Salaya, Nakhon Pathom 73170, Thailand

**Keywords:** epoxy resin, pineapple leaf fiber, cellulose nanofiber, hybrid composite, synergistic toughening

## Abstract

In this work, the effect of cellulose nanofiber (CNF) on the mechanical properties of long pineapple leaf fiber (PALF)-reinforced epoxy composites was investigated. The content of PALF was fixed at 20 wt.% and the CNF content was varied at 1, 3, and 5 wt.% of the epoxy matrix. The composites were prepared by hand lay-up method. Comparison was conducted between CNF-, PALF- and CNF–PALF-reinforced composites. It was found that the introduction of these small amounts of CNF into epoxy resin caused very small effects on flexural modulus and strength of neat epoxy. However, impact strength of epoxy with 1 wt.% CNF increased to about 115% that of neat epoxy, and, as the content of CNF increased to 3 and 5 wt.%, the impact strength decreased to that of neat epoxy. Observation of the fractured surface under electron microscope revealed the change in failure mechanism from a smooth surface to a much rougher surface. For epoxy containing 20 wt.% PALF, both flexural modulus and strength increased significantly to about 300% and 240% that of neat epoxy. The composite impact strength increased to about 700% that of the neat epoxy. For hybrid systems containing both CNF and PALF, there were few changes observed in both flexural modulus and strength compared to the PALF epoxy system. However, much improvement in impact strength was obtained. By using epoxy containing 1 wt.% CNF as the matrix, the impact strength increased to about 220% that of 20 wt.% PALF epoxy or 1520% that of neat epoxy. It thus could be deduced that the spectacular improvement in impact strength was due to the synergistic effect of CNF and PALF. The failure mechanism leading to the improvement in impact strength will be discussed.

## 1. Introduction

A global crisis is driving industries to reduce non-sustainable products and find alternative resources [1,2]. As every industry tries to decease its use of fossil-fuel-based materials, there is more scrutiny around glass fibers [3]. The economic stakes are high enough to consider their replacement due to their end-of-life management, the rising cost of fossil fuel, and their potential to become obsolete, pushing composite industries towards the use of natural fibers [4]. Natural fibers composite has been used in various applications due to its lower costs, weight, environmental impacts, and high carbon storage capacity [5,6]. Shifting from carbon to flax fiber for rotor blades has been reported to save 50% in cumulative energy demand and 45% in greenhouse gas emission (GHG) [7]. The replacement of commercial ABS for car side doors with hemp fiber/epoxy resulted in a decrease of 27% in weight and 15% in GHG emissions [7]. A conventional interior part (37% glass fiber/PP) produced from 50% kenaf/polyhydroxybutyrate could save 0.3 kg CO_2_ equivalent [8]. Although the total life cycle emission decreased, these fiber composites produced higher nitrogen and phosphorus due to fertilizer usage. This highlights the opportunity for the utilization of agricultural waste as a natural reinforcement.

Numerous natural fibers, such as flax, hemp, sisal, and pineapple leaf fibers (PALF), have been evaluated as alternative materials for the construction of green buildings [5,9]. Agricultural waste fiber, such as rice husk, sugarcane, and PALF, is easy to scale up and cost-effective since some of them generate 10–15 times more yield than the actual products [9]. PALF has captured our attention because Thailand is renowned as an agricultural country, which is regarded as one of the top five world’s largest pineapple producers with about 182,902 acres of cultivation area [10]. There are excessive amounts of pineapple leaves left behind after the fruits have been harvested. The byproduct is cheap and has lower life cycle emissions compared to other cultivated plant fibers, such as flax, hemp, and kenaf, which required land, water, and fertilizer. Moreover, PALF has high mechanical properties (34.5–82.5 GPa) [11], which is higher than other cultivated natural fibers, such as flax (58 GPa) [4], hemp (35 GPa) [12], and sisal (9.4–22 GPa) [13]. In addition, the specific modulus of PALF could be competitive with that of glass fiber (72 GPa) [4,12] owing to its inherent properties of high cellulose content [14] and relatively low microfibrillar angle.

Many studies have reported potential utilizations of PALF in various types of polymer composite [13], such as natural rubber [15], nitrile rubber [16], nylon [17], polyamide [18], and epoxy resin [19]. Therefore, PALF can be value-added as a green and promising reinforcement for the future. However, previous studies have mostly evaluated its properties regarding short fiber and non-woven fiber. In this work, continuous PALF was investigated to understand how it could promote higher stress transfer along the composite phase and optimize fiber–matrix properties [12,19].

Currently, nanofiller either from synthetic or natural sources draws a great deal of attention compared with bulk counterparts [20]. As the most abundant, biocompatible, and possessing high mechanical strength similar to that of steel or Kevlar (100–160 GPa) [21], nanocellulose is considered as a good candidate [22,23]. The incorporation of nanofibers greatly increases a material’s tensile strength five-fold and its toughness ten-fold [24] via multiple mechanisms [23,25]. However, nanocellulose has a relatively large carbon footprint [8,26] and could only be added in a small percentage [27,28].

Epoxy resins have been recognized as the most widely used thermoset for manufacturing fiber composite. Bio-based epoxy also provides an opportunity to originate lower-carbon-footprint materials [29]. With the concept of hybridization, different types of interesting components could be combined with epoxy to extend their application in several fields [27,30]. For example, the toughness of carbon-fabric-reinforced EP was increased by 37% after combining modified sugar cane bagasse pulp [31]. The dynamic mechanical properties of nano 3%OPEFB/kenaf/EP and 3%MMT/kenaf/EP hybrid composites increased compared with kenaf/EP [27,32].

In this work, we demonstrate that CNF and PALF could be combined, resulting in synergistic hybrid reinforcement for the epoxy resin, enhancing the impact strength of the composite materials. Fractography was used to understand the mechanism behind this synergistic toughening effect.

## 2. Materials and Methods

### 2.1. Materials and Chemicals

Aqueous dispersion of CNF utilized in this work was prepared from wood pulp, and it is commercially available through Pheedoo Co., Ltd., Bangkok, Thailand. Long PALF was supplied by a local producer in Ratchaburi, Thailand. The long fiber was extracted from fresh pineapple leaf using a decorticating machine. Commercial epoxy resin (EP089) and hardener (HP089) were purchased from a local supplier (Concrete Composite Co., Ltd., Thailand). The matrix was prepared by mixing the resin and hardener at the ratio of 2:1 as recommended by the supplier. Sodium hydroxide (anhydrous 99.5% min on dry basis, Thailand) and commercial-grade acetone (RCI labscan, Thailand) were used without purification.

### 2.2. Preparation of CNF Epoxy Composite (CNF-EP)

To prepare CNF-EP composite, water in the CNF dispersion was first exchanged with acetone to avoid nanofiller aggregation in the dry state. The procedure followed that reported by Tang, et al. [33] with slight modification. Briefly, 1000 mL of acetone was added into the mixture and left for a day; the mixture was then centrifuged at 2500 rpm for 15 min (Sorvall RC 6+, Thermo scientific, Porton Down, UK.), the solvent was decanted, followed by adding acetone to the CNF dispersion. The process was repeated for one week to make sure that all the water had been removed. Solid content of CNF increased from about 4.28% (*w*/*v*) in the aqueous dispersion to about 7.94% (*w*/*v*) after acetone exchange process. The prepared CNF acetone dispersion was diluted to 1.0 wt.% using ultrasonic treatment (BANDELIN, UW 2200, Berlin, Germany) with 70% power for 30 min. Then, epoxy resin HP089 was added into CNF solution. All acetone was eliminated using hot plate stirrer (60 °C) under air ventilation for 24 h and further evaporated in a hot air oven (80 °C) for 72 h. After that, the hardener was gently mixed with the previous epoxy resin and poured into silicone mold. The nanocomposites were left to cure at room temperature for 24 h. The whole process is shown in Figure S1. For neat epoxy composite, epoxy was gently mixed with hardener without CNF in a similar manner and left at room temperature for 24 h. Composites with CNF concentrations of 1, 3, and 5 wt.% were prepared and designated as 1CNF, 3CNF, and 5CNF, respectively.

### 2.3. Preparation of PALF-Reinforced Epoxy Composite (PALF-EP)

**Figure 1:**
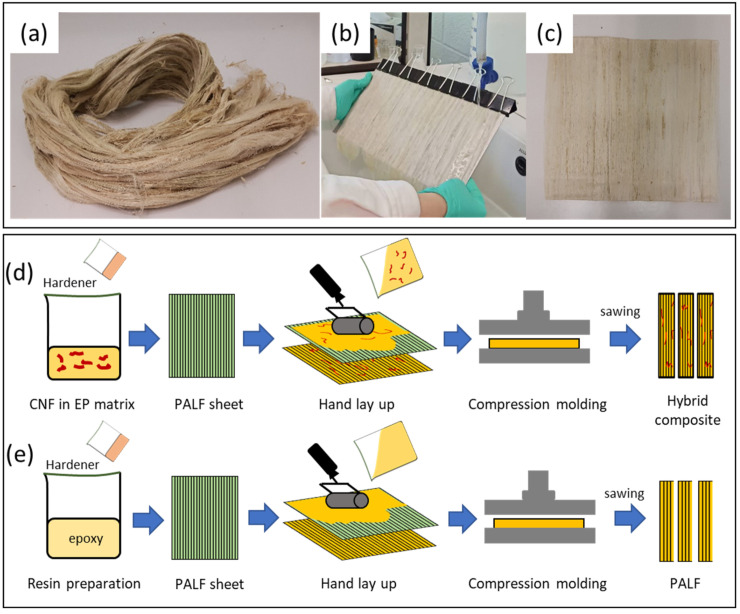
Images of (**a**) pristine PALF, (**b**) PALF alignment under running water, (**c**) dried PALF sheet, and (**d**,**e**) illustrations of composite preparations.

Long pineapple leaf fiber (PALF), which could be regarded as continuous fiber for the specimen size used in this work, was prepared into a well-aligned sheet before incorporated into epoxy resin as shown in Figure 1. Briefly, the fibers were combed to remove unwanted contaminations, untangle, and straighten all the fibers. After that, approximately 20 g of the fiber was immersed in 1 L of 10 wt.% sodium hydroxide solution for 24 h and then washed several times with tap water until the pH of the water equaled 7. The fibers were carefully arranged under running water to create a well-aligned sheet of fibers, which was then dried in a hot air oven at 60 °C. The sheet was cut into appropriate size and impregnated with a mixture of epoxy/hardener or epoxy/hardener/CNF. After that, the epoxy-impregnated PALF sheets were subsequently laid down in a closed mold and left at room temperature to cure for 24 h. The composite obtained was about 3 mm thick and contained 20 wt.% PALF.

### 2.4. Characterization

#### 2.4.1. Electron Microscopy (EM)

Transmission electron microscope (JEOL, JSM-1400, Tokyo, Japan) was used to observe the size and shape of cellulose nanofiber (CNF). To prepare the sample for the analysis, CNF suspension was diluted in distilled water to concentration of about 0.01 wt.% and sonicated for 30 min. A small drop of the suspension was placed on 300-mesh-Formvar-coated copper grids and allowed to dry at room temperature. The samples were stained with iodine vapor to improve image contrast before observation at an operating voltage of 100 kV.

Field emission scanning electron microscope (JEOL, JSM-7610FPlus, Tokyo, Japan) was used to study the fracture surface of the composite after flexural and impact testing at room temperature. The samples were initially dried in the oven (80 °C) and mounted on SEM holder using double-sided carbon adhesive tape to achieve the appropriate conductivity. After that, the samples were coated with a thin layer of titanium (JEOL, JEC-3000FC, Tokyo, Japan) to prevent surface damaging during the operation at 5 kV.

#### 2.4.2. Differential Scanning Calorimetry (DSC)

Glass transition temperature of the epoxy matrix was determined on a differential scanning calorimeter (DSC Q-200, TA Instrument, New Castle, USA). The transition temperature was determined from the first heating cycle from 0 °C to 150 °C with a heating rate of 10 °C/min under nitrogen atmosphere.

#### 2.4.3. Mechanical Characterization

Flexural test: The test was carried out on a universal testing machine (Instron 5569, Norwood, Massachusetts, USA) following ASTM D790-3 using a crosshead speed of 1.28 mm/min, 5 kN of load cell, and a support span length of 48 mm. Specimens were cut from composite sheets with a scroll saw into strips of 12.7 mm wide and 120 mm long with the long axis parallel to the fiber direction. The average values of flexural strength and flexural modulus from 5 specimens were reported.

Impact test: The test was carried out on a pendulum impact testing machine (Zwick/Roell HIT5.5P, Ulm, Germany) in Izod configuration following ASTM D256. The composite samples were cut with a scroll saw from composite sheets along the fiber direction, providing a strip 12.7 mm wide and 60 mm long. The samples were notched with a Zwick/Roell manual notch cutting machine. Average values of 5 specimens were reported.

### 2.5. Statistical Analysis

Statistical analysis was performed using analysis of variance (ANOVA) and Data Analysis tool in Microsoft Excel (Office16) program. The *t*-test method, using Two-Sample Assuming Unequal Variances, was performed to analyze differences among the means at a confidence level of 95%.

## 3. Results and Discussion

### 3.1. Some Characteristics of CNF and PALF

**Figure 2:**
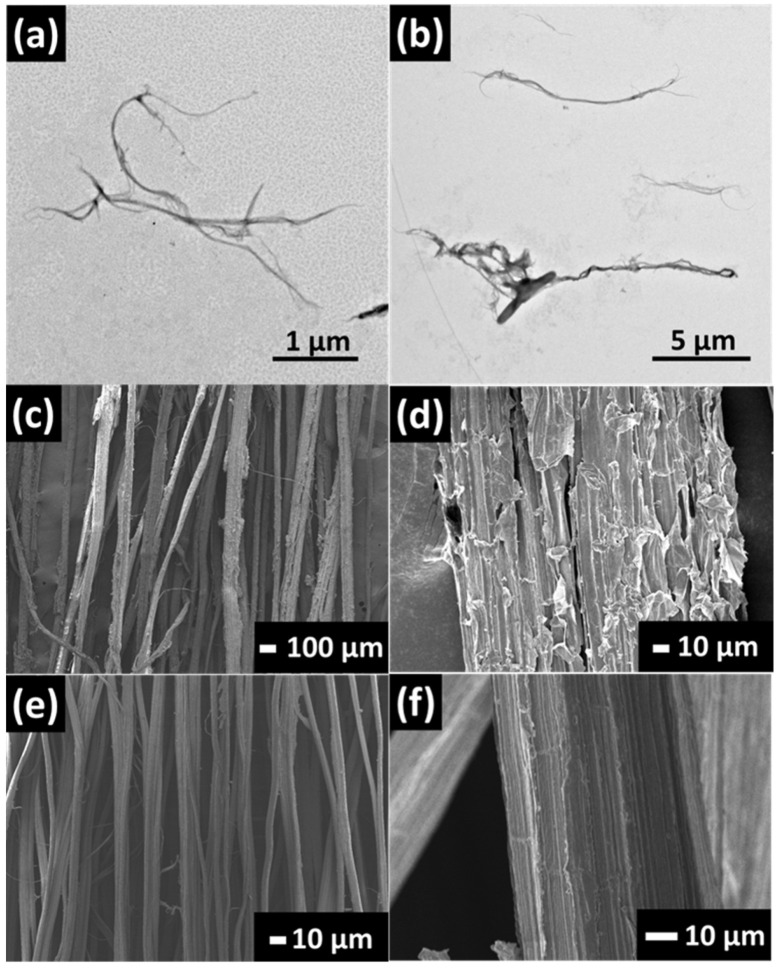
(**a**,**b**) TEM images of CNF, FESEM images of (**c**,**d**) untreated pineapple leaf fiber, and (**e**,**f**) PALF after alkaline treatment.

Figure 2a,b displays the TEM images of individual CNF. CNF has a long fibrillar shape with diameters in the range of 25 to 125 nm. The length of CNF was around 9.8 ± 3.6 µm. However, the exact length of CNF was difficult to determine folding and overlapping of individual nanofibers. Figure 2c–f clearly displays the surface of PALF before and after alkaline treatment. Starting PALF consists of microfibers held together to form bundles of varying sizes, presumably with hemicellulose [34]. When washed with sodium hydroxide solution, the fiber surface became smoother and size reduced from around 52 ± 28 µm to approximately 44 ± 23 µm. Sodium-hydroxide-treated PALF was used for the rest of the work for composite preparation.

### 3.2. Structural Analysis of the Matrix with DSC

In certain cases, addition of nanomaterial could affect the network structure of the epoxy matrix [35,36] and thus its T_g_ and other properties. Therefore, DSC was investigated if CNF had such an effect in the system under investigation. Figure S2 displays thermograms of epoxy filled with different amounts of CNF. Some thermograms showed obscure and unusual baseline change instead of a single and clear step change in the baseline. Thus, it is difficult to determine the change in the baseline. Despite that difficulty, there is only slight variation in the glass transition temperature, T_g_, for most systems (Table 1). It should be noted that, for 1CNF, T_g_ dropped by about 9 °C and increased, converging to the T_g_ of neat epoxy with increasing amount of CNF. For 20PALF, T_g_ increased by about 4 °C; no appreciable change was observed with the addition of CNF into the matrix. The increase is about the same order as that observed in 20 wt.% microfibrillated filled epoxy [33]. In addition, the T_g_ values of these systems were determined using DMA and it was found that their T_g_ values were very similar (see Supplementary Materials) but somewhat higher than those obtained from DSC method [37]. This suggests greater interactions, namely physical and chemical interactions, occurring between PALF and the epoxy network. Thus, it may be concluded that the matrices in all systems have comparable network structures.

### 3.3. Flexural Properties

**Figure 3:**
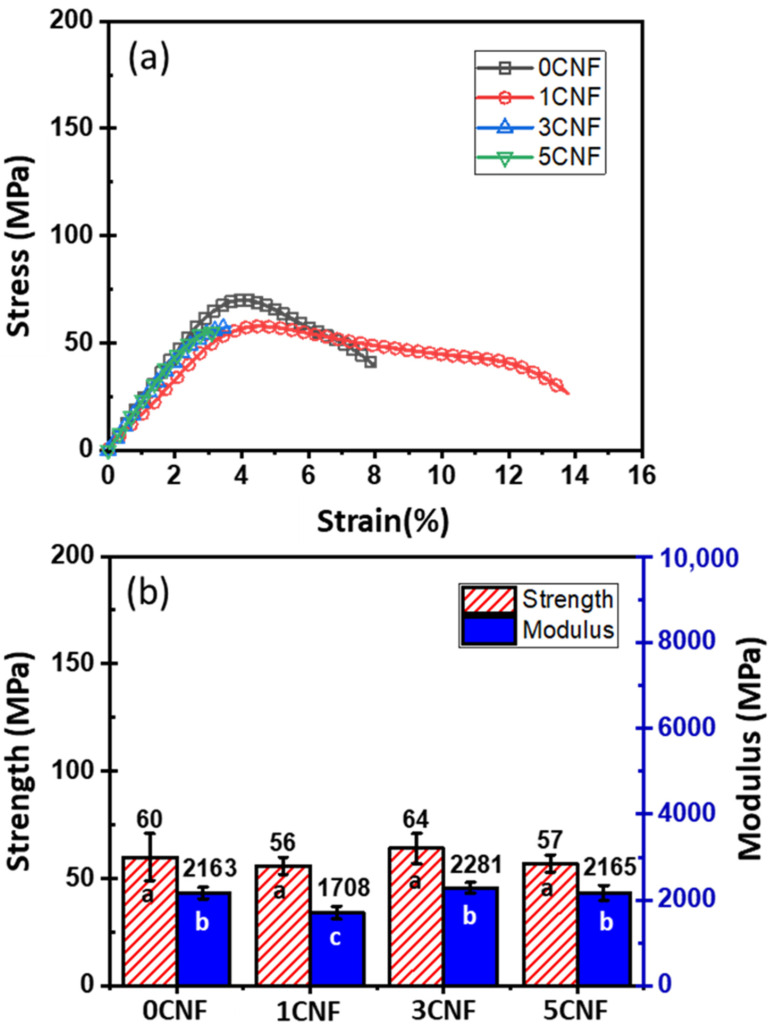
(**a**) Stress–strain curves and (**b**) average strengths and moduli at 1% strain of neat epoxy and CNF-filled epoxy composites. Different letters on each bar indicate statistically significant differences in the means.

Flexural test was used to determine the mechanical properties of the composites, and representative stress–strain curves for CNF-filled epoxy systems are shown in Figure 3a. The average values for maximum stress (i.e., strength) and moduli at 1% strain of CNF-filled epoxy composites containing different contents of CNF are shown in Figure 3b. The composite with 1 wt.% CNF shows slight reduction in both strength and modulus with an appreciable increase in failure strain at around 8% to 14%. This is probably related to the reduction in T_g_ shown previously (see Table 1). With increasing CNF content (3 and 5 wt.%), flexural strength and modulus remain virtually the same while failure strain drops significantly from about 8% to 3%. These results indicate that the CNF epoxy nanocomposites become more brittle at higher levels of CNF loadings (>1 wt.%), and no improvement in strength and modulus can be gained with this relatively low range of CNF content.

**Figure 4:**
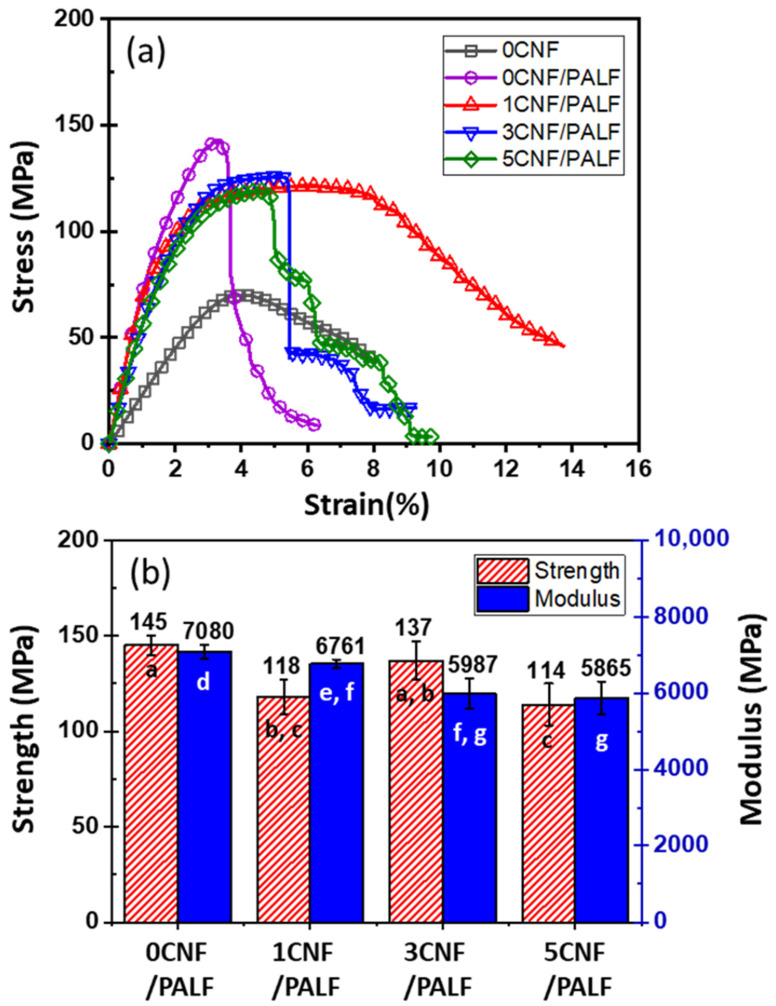
(**a**) Stress–strain curves and (**b**) average strengths and moduli at 1% strain of 20PALF in different matrices. Different letters on each bar indicate statistically significant differences in the means.

The above epoxy systems were used as the matrix material to prepare the composite material with 20 wt.% PALF. The stress–strain curves of these composites are displayed in Figure 4a. The stress–strain curve for neat epoxy is included for the purpose of comparison. PALF causes a significant increase in the stress, but the composite fails at lower strain. When the matrix was changed to 1CNF, 3CNF, and 5CNF, the slopes of the curves and maximum stresses dropped slightly, while the failure strains increased significantly and then decreased. The failure strains of 3CNF and 5CNF composites are, however, still higher than that of 0CNF. Average values for flexural strengths and moduli of the composites are shown in Figure 4b. The pattern of change in these flexural properties reflects the change in the content of the matrix material.

### 3.4. Impact Property

**Figure 5:**
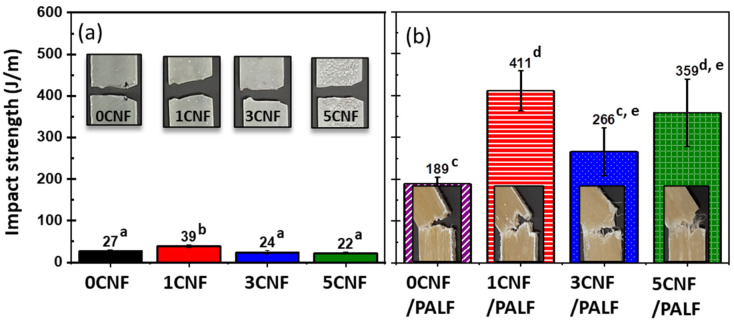
Impact strengths of (**a**) CNF-reinforced epoxy composite and (**b**) CNF/PALF in different matrices. Different letters on each bar indicate statistically significant differences in the means.

The ability of a material to withstand a high force or shock applied over a short time period is known as impact strength (or otherwise known as toughness). This can be measured as impact strength. Figure 5 displays the impact strengths of CNF-filled epoxy composites and hybrid composites. With regard to the effects of CNF, it is observed that the addition of 1 wt.% CNF resulted in an increase in impact strength (by 44%) from 27 J/m to 31 J/m. When CNF was added to 3 and 5 wt.%, the impact strengths decreased slightly to 22 and 25 J/m, respectively.

When PALF was added to epoxy (0CNF/PALF), the impact strength increased (by 600%) from 27 J/m to 189 J/m compared to the neat epoxy. This increase in impact strength could be contributed by both crack deflection and de-bonding mechanisms at the fiber–matrix interface. When epoxy was replaced with 1CNF in a hybrid system (1CNF/PALF), the strength increased to 411 J/m, which is approximately 220% that of the system without CNF. However, when the amount of CNF was increased to 3 and 5 wt.%, the impact strengths decreased but were still higher than that of the system without CNF. This decrease was in good agreement with what was observed previously for CNF-filled epoxy composites. These results clearly reveal the synergistic toughening effect of hybrid CNF and PALF that provides higher toughness as compared to the single reinforcement system.

### 3.5. Fractography

**Figure 6:**
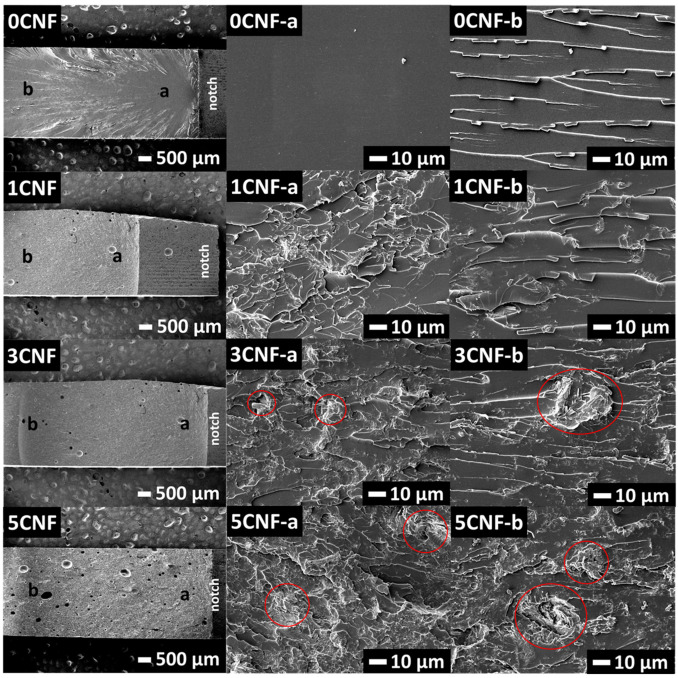
SEM images of impacted specimens of CNF-filled epoxy composites at low magnification (**left**) and high magnification of corresponding regions (a, b) in the images (panels on the **left** column). Crack propagated from the right side of the specimen as indicated by notch in low magnification images (left column) to the left side. For areas in red circles see main text.

In order to understand the mechanism regulating the improvement in impact strength of the hybrid CNF–PALF epoxy composites, impacted fracture surfaces were studied. The impact fracture surfaces of epoxy filled with CNF are shown in Figure 6. For each composite, the overall view is shown and two areas, which are labeled ‘a’ and ‘b’, are shown at high magnification. Neat epoxy or 0CNF displays a typical brittle fracture surface; i.e., the area close to notch ‘a’ is smooth and featureless, and, in areas further away from notch ‘b’, cracks deflection and branching are observed. With the addition of CNF, the fracture surfaces become much rougher over the whole area. Crack deflection and branching start right at the notch. The roughness increases with increasing CNF content. Large CNFs are clearly observed protruding from the surface (red circles in Figure 6). Although this crack deflection mechanism [30] and craze-like region has been linked to massive energy absorption [38], little change in impact energy was observed (see Figure 5a). Only a slight increase is observed for the 1CNF, and the impact strength drops to values slightly lower than that of neat epoxy. This is probably due to the presence of voids and large filler aggregation that are not effective in retarding crack propagation through the matrix [39,40].

**Figure 7:**
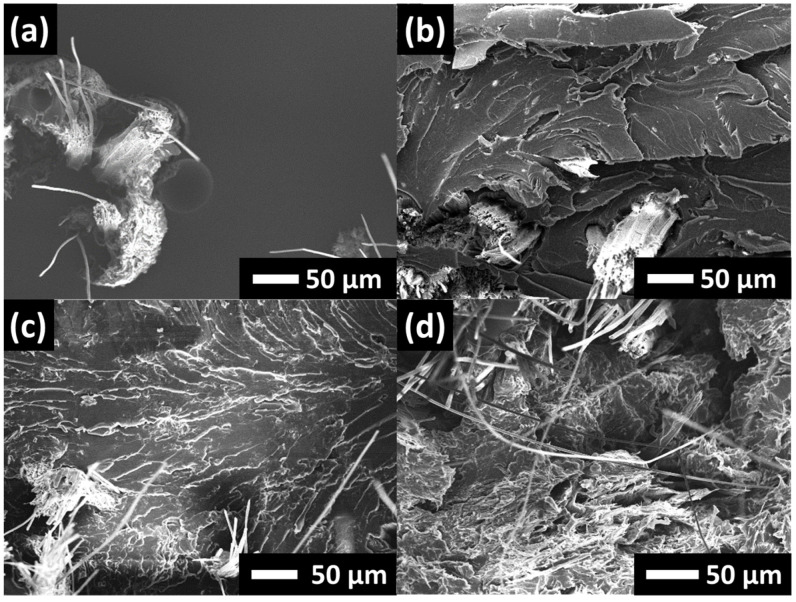
Fractographic images of impacted specimens of 20PALF in different matrices: (**a**) neat EP, (**b**) 1CNF, (**c**) 3CNF, and (**d**) 5CNF. Scale bars: 50 µm.

The impact fracture surfaces of hybrid composites are shown in Figure 7. When the areas between large bungles of PALF are considered, similar patterns of fracture surfaces to that of CNF-filled epoxy are observed. In the absence of CNF, the fracture surfaces were observed to be relatively smooth. When the CNFs were added into the epoxy, the fracture surfaces yielded very rough features; this reflects an increase in fracture surface areas and indicates that more fracture energy was needed to break the composite material [41].

### 3.6. Possible Toughening Mechanism

**Figure 8:**
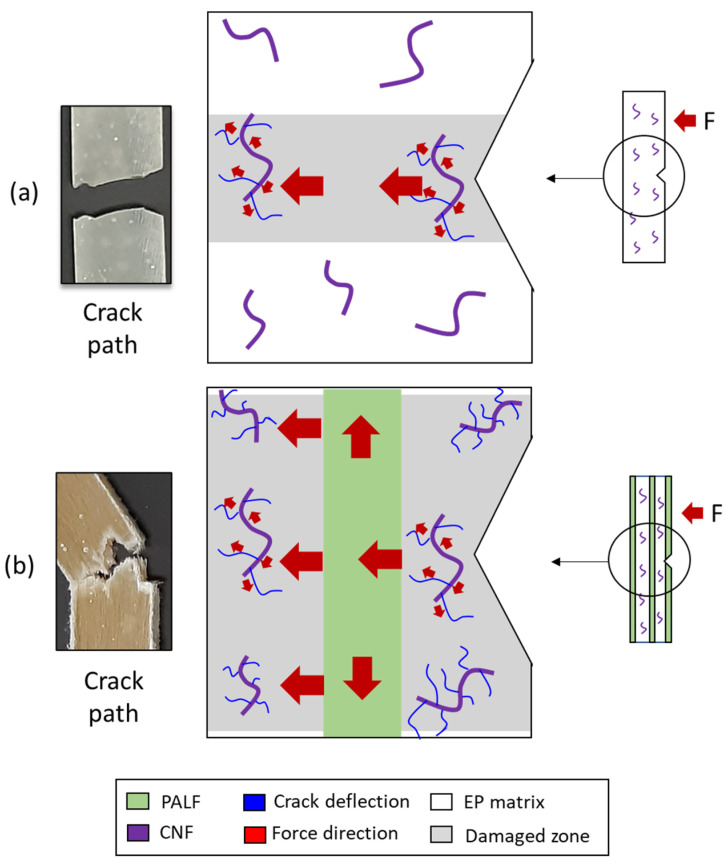
Possible reinforcing mechanism after impact loading of (**a**) CNF-reinforced EP and (**b**) hybrid epoxy composite (CNF/PALF).

In the previous section, the impact fracture surfaces of CNF-filled epoxy composites and those of hybrid composites were compared and not much could be deduced except that multiple crack deflections operate in both systems. One important point to note is that crack paths in CNF-filled and hybrid composites are very different, and the latter is much more complicated. Broken surfaces of hybrid composites are very rough and have a large number of protruding PALFs. This indicates that stress could be transferred along the fiber to a much greater volume of materials, causing a much greater number of crack deflection points within such volume and in so doing absorbing a substantial amount of energy. The mechanism can be schematically presented in Figure 8.

In the case of the CNF-reinforced EP composite material (Figure 8, top panel), when an external load is applied in the direction as indicated, the EP matrix deforms. The deforming matrix shears over the interface between the EP and CNF, causing shear stress to be generated at the interface. This in turn caused the CNF to bend, and bending stress is generated. For a sufficiently high external load, the interfacial shear stress peaks at the ends of the CNF and this leads to matrix yielding and finally cracking; i.e., cracks are initiated in the matrix adjacent to the CNF ends [42]. The cracks propagate into the matrix. In the absence of PALF, i.e., the stronger phase (compared to the EP matrix), there is nothing standing in the way of the path of the crack propagation. In other words, the crack propagation could not be halted [42]. The crack propagates cleanly throughout the specimen cross section until the specimen fractures into two.

In the case of the hybrid (CNF/PALF-reinforced) epoxy composite material (Figure 8, bottom panel), in the presence of the external load, the EP matrix deforms. The deforming matrix shears over the interface between the EP and CNF, causing shear stress to be generated at the interface. This in turn caused the CNF to bend, and bending stress is generated. In addition, the deforming matrix also compresses the interface between the EP and PALF. Compressive stresses generated at the interface lead to bending of the PALF. Thus, both the PALF and CNF take up stress from the matrix in this way. For a sufficiently high external load, similar to the previous case, the matrix crack appears as the CNF end propagates, but on reaching the PALF, the propagation is halted [42]. If the load is much higher, eventually, the stress taken up by the PALFs (as they all bend) could exceed the fracture strength of the PALF, and thus the PALFs break. The crack in matrix material continues to propagate past the broken ends of the PALFs, leading to a complete fracture of the composite material.

## 4. Conclusions

Hybrid epoxy composites with improved mechanical properties were successfully prepared from CNF and PALF. The technique offers great enhancements in stiffness, strength, and toughness to the epoxy matrix. Being very small, incorporation of CNF is limited to very low loading, yet the amount of only 1 wt.% was found to be very effective in improving the impact strength while having very little effect on flexural properties. This is due to the ability of CNF to change the failure mechanism, at high speed, of the epoxy matrix. The PALF, on the other hand, allows much greater content to be incorporated with ease, and, hence, both flexural properties and impact could be significantly improved. By combining both CNF and PALF together, spectacular improvement in toughness is obtained. This is a result of the synergistic effect of CNF and PALF. This could lead to greener, more sustainable, and more cost-effective materials for demanding applications.

## Figures and Tables

**Table 1:** Glass transition temperature (T_g_) of nanocomposite reinforced with different CNF contents determined with DSC.

Composite System	T_g_ Onset	T_g_ (°C)
0CNF	47	55
1CNF	43	49
3CNF	46	52
5CNF	51	55
0CNF/PALF	56	59
1CNF/PALF	54	59
3CNF/PALF	53	56
5CNF/PALF	56	59

## Data Availability

The data presented in this study are available on request from the corresponding author.

## Supplementary Materials

The following supporting information can be downloaded at: https://www.mdpi.com/article/10.3390/nano13111703/s1, Figure S1: (a) Preparation of cellulose nanofiber reinforced epoxy composite (CNF/EP), (b) images of solution of CNF before and after ultrasonic treatment, (c) images of 1CNF, 3CNF, and 5CNF before hardening addition; Figure S2: DSC curves of (a) 0CNF (b) 1CNF (c) 3CNF (d) 5CNF (e) 0CNF/PALF (f) 1CNF/PALF (g) 3CNF/PALF (h) 5CNF/PALF; Figure S3: Tan delta of the nanocomposites resulted from DMA analysis; Table S1: Glass transition temperature of nanocomposites.

## References

[1] Ishii J., Osuga M., Okada T., Miyazaki H., Koseki M., Tanikoshi K. (2008). Reduction of CO_2_ Emissions for Automotive Systems. Hitachi Revi.

[2] Dufluo J.R., Deng Y., Acker K.V., Dewulf W. (2012). Do fiber-reinforced polymer composites provide environmentally benign alternatives? A life-cycle-assessment-based study. MRS Bulletin.

[3] Sarikaya E., Çallioğlu H., Demirel H. (2019). Production of epoxy composites reinforced by different natural fibers and their mechanical properties. Compos. B. Eng..

[4] Poilâne C., Cherif Z.E., Richard F., Vivet A., Ben Doudou B., Chen J. (2014). Polymer reinforced by flax fibres as a viscoelastoplastic material. Compos. Struct..

[5] Faruk O., Bledzki A.K., Fink H.-P., Sain M. (2014). Progress Report on Natural Fiber Reinforced Composites. Macromol. Mater. Eng..

[6] Mathijsen D. (2018). The renaissance of flax fibers. Reinf. Plast..

[7] De Vegt O.M., Haije W.G. (1997). Comparative Environmental Life Cycle Assessment of Composite Materials.

[8] Boland C.S., De Kleine R., Keoleian G.A., Lee E.C., Kim H.C., Wallington T.J. (2016). Life Cycle Impacts of Natural Fiber Composites for Automotive Applications: Effects of Renewable Energy Content and Lightweighting. J. Ind. Ecol..

[9] Sangmesh B., Patil N., Jaiswal K.K., Gowrishankar T.P., Selvakumar K.K., Jyothi M.S., Jyothilakshmi R., Kumar S. (2023). Development of sustainable alternative materials for the construction of green buildings using agricultural residues: A review. Constr. Build. Mater..

[10] (2022). Deputy Permanent Secretary of the Ministry of Agriculture and Cooperatives Sits at the Head of the Table for the Meeting of the National Pineapple Policy and Development Committee to Consider the Pineapple Development Plan for the Years 2023–2027. https://shorturl.at/ksEFR.

[11] Kengkhetkit N., Amornsakchai T. (2012). Utilisation of pineapple leaf waste for plastic reinforcement: A novel extraction method for short pineapple leaf fiber. Ind. Crops. Prod..

[12] Oksman K. (2016). High Quality Flax Fibre Composites Manufactured by the Resin Transfer Moulding Process. J. Reinf. Plast. Compos..

[13] Mishra S., Mohanty A.K., Drzal L.T., Misra M., Hinrichsen G. (2004). A Review on Pineapple Leaf Fibers, Sisal Fibers and Their Biocomposites. Macromol. Mater. Eng..

[14] Ahmed M.M., Dhakal H.N., Zhang Z.Y., Barouni A., Zahari R. (2021). Enhancement of impact toughness and damage behaviour of natural fibre reinforced composites and their hybrids through novel improvement techniques: A critical review. Compos. Struct..

[15] Surajarusarn B., Hajjar-Garreau S., Schrodj G., Mougin K., Amornsakchai T. (2020). Comparative study of pineapple leaf microfiber and aramid fiber reinforced natural rubbers using dynamic mechanical analysis. Polym. Test..

[16] Wisittanawat U., Thanawan S., Amornsakchai T. (2014). Remarkable improvement of failure strain of preferentially aligned short pineapple leaf fiber reinforced nitrile rubber composites with silica hybridization. Polymer Testing.

[17] Nopparut A., Amornsakchai T. (2016). Influence of pineapple leaf fiber and it’s surface treatment on molecular orientation in, and mechanical properties of, injection molded nylon composites. Polymer Testing.

[18] Panyasart K., Chaiyut N., Amornsakchai T., Santawitee O. (2014). Effect of Surface Treatment on the Properties of Pineapple Leaf Fibers Reinforced Polyamide 6 Composites. Energy Procedia.

[19] Jain J., Jain S., Sinha S. (2018). Characterization and thermal kinetic analysis of pineapple leaf fibers and their reinforcement in epoxy. J. Elastomers Plast..

[20] Chakrabarty A., Teramoto Y. (2018). Recent Advances in Nanocellulose Composites with Polymers: A Guide for Choosing Partners and How to Incorporate Them. Polymers.

[21] Lee H.-J., Lee H.-S., Seo J., Kang Y.-H., Kim W., Kang T. (2019). State-of-the-Art of Cellulose Nanocrystals and Optimal Method for their Dispersion for Construction-Related Applications. Appl. Sci..

[22] Lee K.-Y., Aitomäki Y., Berglund L.A., Oksman K., Bismarck A. (2014). On the use of nanocellulose as reinforcement in polymer matrix composites. Compos. Sci. Technol..

[23] Kim J.-H., Shim B.S., Kim H.S., Lee Y.-J., Min S.-K., Jang D., Abas Z., Kim J. (2015). Review of nanocellulose for sustainable future materials. Int. J. Precis. Eng. Manuf..

[24] Rudich A., Sapru S., Shoseyov O. (2023). Biocompatible, Resilient, and Tough Nanocellulose Tunable Hydrogels. Nanomaterials.

[25] Xu X., Liu F., Jiang L., Zhu J.Y., Haagenson D., Wiesenborn D.P. (2013). Cellulose nanocrystals vs. cellulose nanofibrils: A comparative study on their microstructures and effects as polymer reinforcing agents. ACS Appl. Mater. Interfaces.

[26] Li Q., McGinnis S., Sydnor C., Wong A., Renneckar S. (2013). Nanocellulose Life Cycle Assessment. ACS Sustain. Chem. Eng..

[27] Saba N., Paridah M.T., Abdan K., Ibrahim N.A. (2016). Effect of oil palm nano filler on mechanical and morphological properties of kenaf reinforced epoxy composites. Constr Build Mater..

[28] Saba N., Safwan A., Sanyang M.L., Mohammad F., Pervaiz M., Jawaid M., Alothman O.Y., Sain M. (2017). Thermal and dynamic mechanical properties of cellulose nanofibers reinforced epoxy composites. Int. J. Biol. Macromol..

[29] Vinod A., Sanjay M.R., Siengchin S., Fischer S. (2021). Fully bio-based agro-waste soy stem fiber reinforced bio-epoxy composites for lightweight structural applications: Influence of surface modification techniques. Constr. Build. Mater..

[30] Alamri H., Low I.M. (2012). Characterization of epoxy hybrid composites filled with cellulose fibers and nano-SiC. J. Appl. Polym. Sci..

[31] Uribe B.E.B., Chiromito E.M.S., Carvalho A.J.F., Arenal R., Tarpani J.R. (2017). TEMPO-oxidized cellulose nanofibers as interfacial strengthener in continuous-fiber reinforced polymer composites. Mater. Des..

[32] Saba N., Paridah M.T., Abdan K., Ibrahim N.A. (2016). Dynamic mechanical properties of oil palm nano filler/kenaf/epoxy hybrid nanocomposites. Constr. Build. Mater..

[33] Tang L., Weder C. (2010). Cellulose whisker/epoxy resin nanocomposites. ACS Appl. Mater. Interfaces.

[34] Surajarusarn B., Traiperm P., Amornsakcha T. (2019). Revisiting the Morphology, Microstructure, and Properties of Cellulose Fiber from Pineapple Leaf so as to Expand Its Utilization. Sains Malaysiana.

[35] Ansari F., Galland S., Johansson M., Plummer C.J.G., Berglund L.A. (2014). Cellulose nanofiber network for moisture stable, strong and ductile biocomposites and increased epoxy curing rate. Compos. Part A Appl. Sci. Manuf..

[36] Jayan J.S., Saritha A., Deeraj B.D.S., Joseph K. (2020). Triblock copolymer grafted Graphene oxide as nanofiller for toughening of epoxy resin. Mater. Chem. Phys..

[37] de Souza J.P.B., dos Reis J.M.L. (2015). A Thermomechanical and Adhesion Analysis of Epoxy/Al_2_O_3_ Nanocomposites. Nanomater. Nanotechnol..

[38] Rattanawijan W., Amornsakchai T., Amornsakchai P., Petiraksakul P. (2009). Influence of compatibilizer on notched impact strength and fractography of HDPE-organoclay composites. J. Appl. Polym. Sci..

[39] Chaturvedi R., Pappu A., Tyagi P., Patidar R., Khan A., Mishra A., Gupta M.K., Thakur V.K. (2022). Next-generation high-performance sustainable hybrid composite materials from silica-rich granite waste particulates and jute textile fibres in epoxy resin. Ind. Crops. Prod..

[40] Agustina E., Goak J.C., Lee S., Kim Y., Hong S.C., Seo Y., Lee N. (2023). Effect of Graphite Nanoplatelet Size and Dispersion on the Thermal and Mechanical Properties of Epoxy-Based Nanocomposites. Nanomaterials.

[41] Choi W.J., Lee S.Y., Park S.J. (2022). Effect of Ambient Plasma Treatments on Thermal Conductivity and Fracture Toughness of Boron Nitride Nanosheets/Epoxy Nanocomposites. Nanomaterials.

[42] Goh K.L. (2017). Discontinuous-Fibre Reinforced Composites.

