# Supplementary material for: Synergistic Toughening of Epoxy Composite with Cellulose Nanofiber and Continuous Pineapple Leaf Fiber as Sustainable Reinforcements
Source: Nanomaterials (Basel). 2023 May 23;13(11):1703. doi: 10.3390/nano13111703 (PMC10254581; doi:10.3390/nano13111703)
Supplement: Supplementary file 1 [file nanomaterials-13-01703-s001.zip › nanomaterials-2406357-supplementary.pdf]

Supplementary materials

Synergistic Toughening of Epoxy Composite with Cellulose Nanofiber and Continuous Pineapple Leaf Fiber as Sustainable Reinforcements

Nichapa Klinthoopthamrong [1], Sombat Thanawan [1,2], Gautier Schrodj [3], Karine Mougin [3], Kheng-Lim Goh [4,5] and Taweechai Amornsakchai [1,6,*]

1 Polymer Science and Technology Program, Department of Chemistry, Faculty of Science, Mahidol University, Phuttamonthon 4 Road, Salaya, Nakhon Pathom 73170, Thailand; sombat.tha@mahidol.ac.th (S.T.)

2 Rubber Technology Research Center, Faculty of Science, Mahidol University, Phuttamonthon 4 Road, Salaya, Nakhon Pathom 73170, Thailand

3 Institut de Science des Matériaux de Mulhouse, IS2M-CNRS-UHA, 15, Rue Jean Starcky, B.P.2488-68057 Mulhouse, Cedex, France; gautier.schrodj@uha.fr (G.S.); karine.mougin@uha.fr (K.M.)

4 Mechanical Design and Manufacturing Engineering, Newcastle University in Singapore, 172A Ang Mo Kio Avenue 8 #05-01, SIT@NYP Building, Singapore 567739, Singapore; kheng-lim.goh@newcastle.ac.uk

5 Faculty of Science, Agriculture & Engineering, Newcastle University, Newcastle upon Tyne NE1 7RU, UK

6 Center of Sustainable Energy and Green Materials, Faculty of Science, Mahidol University, Phuttamonthon 4 Road, Salaya, Nakhon Pathom 73170, Thailand

* Correspondence: taweechai.amo@mahidol.ac.th

Preparation of CNF-epoxy composite (CNF-EP)

The preparation of CNF reinforced epoxy composite is shown in **Figure S1a**. Ultrasonic treatment was applied to facilitate CNF dispersion in acetone media, as show in **Figure S1b.** Moreover, we found that the addition of a higher amount of CNF seriously increased the viscosity of the epoxy resin, as showed in **Figure S1c**. Nanocomposite reinforced with CNF content higher than 1 wt.% caused difficulty in homogenous mixing. Consequently, unavoidable tiny voids were trapped inside the materials, and fiber aggregation occurred. The mechanical properties of the composite reinforced with a higher amount of CNF cloud be diminished.

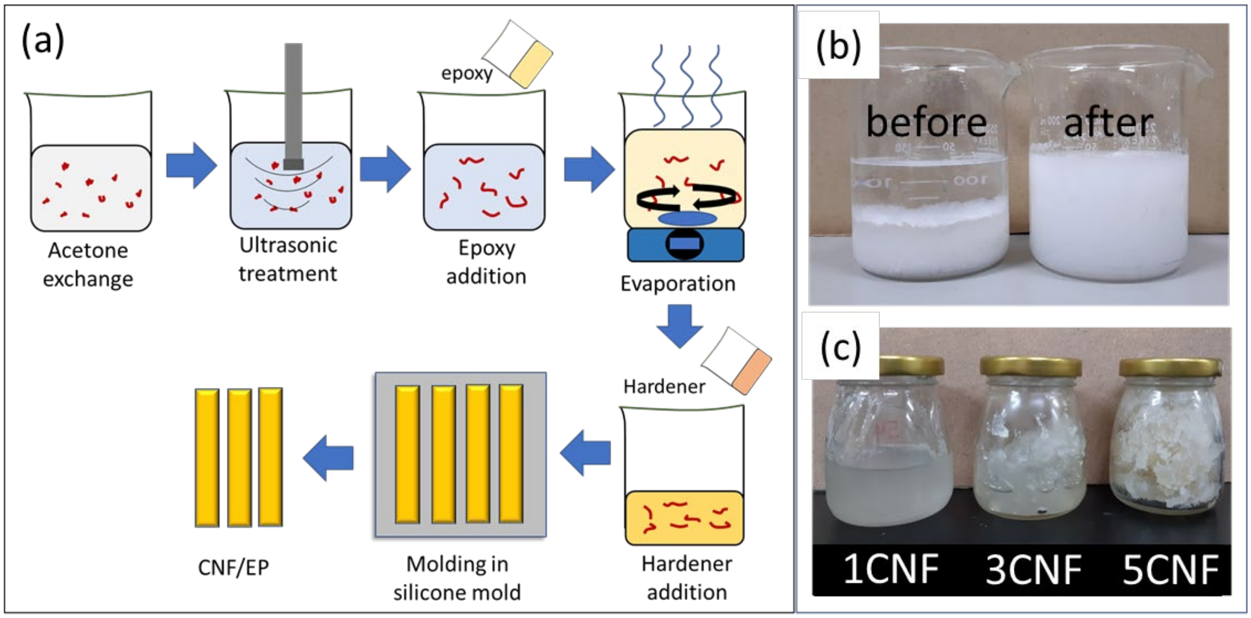

Figure S1. (**a**) Preparation of cellulose nanofiber reinforced epoxy composite (CNF/EP), (**b**) images of solution of CNF before and after ultrasonic treatment, (**c**) images of 1CNF, 3CNF, and 5CNF before hardening addition.

Structural analysis of the matrix with DSC

Figure S2 displays thermograms of epoxy filled with varying amounts of CNF. Some thermograms showed an obscure and unusual baseline change instead of a single, clear step change in the baseline. The glass transition temperature (T_g) was determined by the intersection between the onset temperature and the transition point, and is represented in **Table 1** of the main paper.

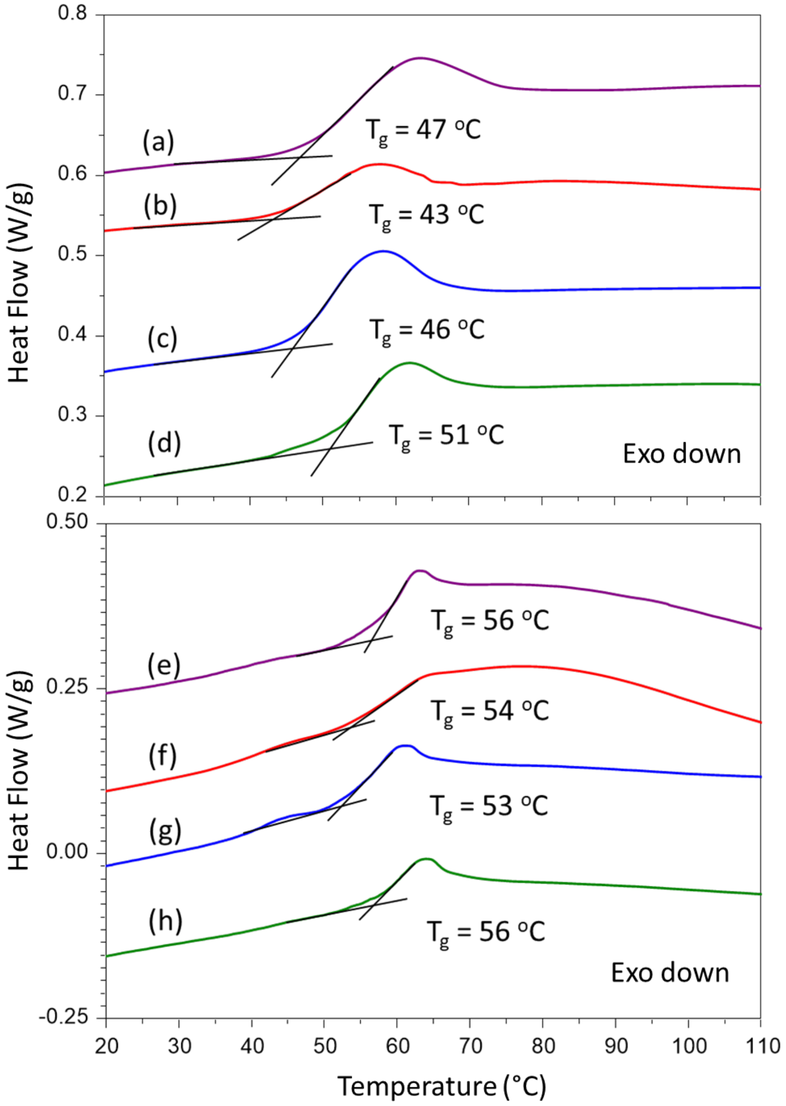

Figure S2. DSC curves of (**a**) 0CNF (**b**) 1CNF (**c**) 3CNF (**d**) 5CNF (**e**) 0CNF/PALF (**f**) 1CNF/PALF (**g**) 3CNF/PALF (**h**) 5CNF/PALF.

Structural analysis of the matrix with DSC

The nanocomposites were characterized in double cantilever mode using a Viscoanalyzer (Metravib VA 4000, Limonest, France). The T_g (**Table S1**) was calculated from the maximum peak of the Tan delta thermogram, as shown in **Figure S3**. We also observed a slight drop in the T_g calculated from DMA analysis of 1CNF. Furthermore, the T_g of the overall nanocomposite did not show a significant difference compared to the previous results obtained from DSC measurement. However, the T_g resulting from the DMA analyzer was somewhat higher than those obtained from the DSC method [1].

Table S1. Glass transition temperature of nanocomposites

Composite system	T_g (°C) from DSC	T_g (°C) from DMA
0CNF	55	69
1CNF	49	67
3CNF	52	69
5CNF	55	69

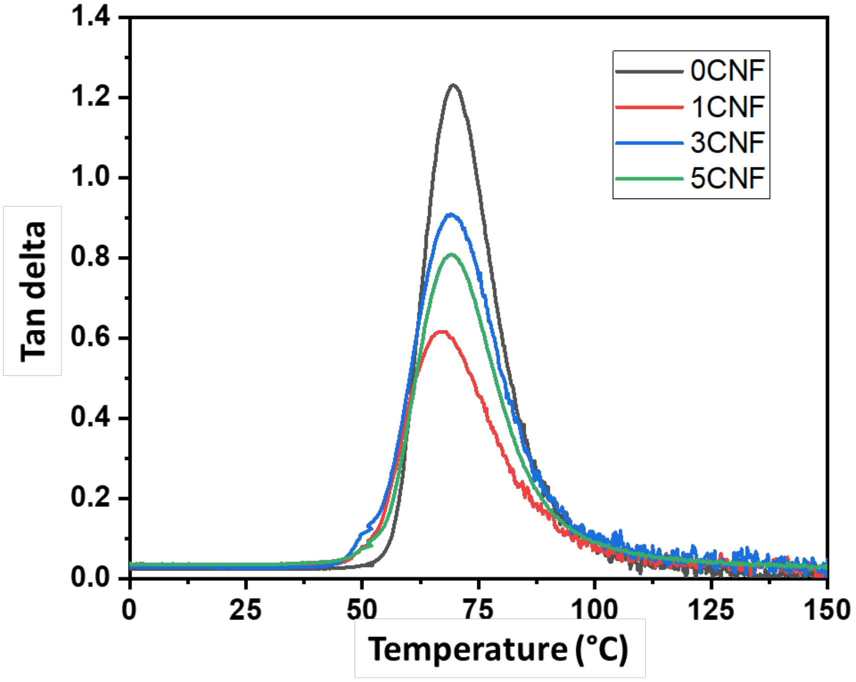

Figure S3. Tan delta of the nanocomposites resulted from DMA analysis.

Reference

1 Souza, J.P.B.d.; Reis, J.M.L.d. A Thermomechanical and Adhesion Analysis of Epoxy/Al2O3 Nanocomposites. *Nanomater. Nanotechnol.* **2015**, *5*, 1-7. DOI: 10.5772/60938.
